# Tetramethylpyrazine attenuates sodium arsenite-induced acute kidney injury by improving the autophagic flux blockade via a YAP1-Nrf2-p62-dependent mechanism

**DOI:** 10.7150/ijbs.104107

**Published:** 2025-01-13

**Authors:** Zhiyong Song, Tom K. Hei, Xuezhong Gong

**Affiliations:** 1 Department of Nephrology, Shanghai Municipal Hospital of Traditional Chinese Medicine, Shanghai University of Traditional Chinese Medicine, 274 Zhijiang Middle Road, Shanghai 200071, China.; 2 Center for Radiological Research, College of Physician and Surgeons, Columbia University, 630 West 168th Street, NY 10032, USA.

**Keywords:** tetramethylpyrazine, sodium arsenite, acute kidney injury, autophagic flux blockade, YAP1

## Abstract

With increased application, sodium arsenite (AS III)-induced acute kidney injury (AI-AKI) is becoming a new clinical challenge, but its potential pathogenesis remains poorly studied. Our previous data demonstrated that inducing autophagy and mitochondrial dysfunction in renal tubular cells are important links of AI-AKI and could be inhibited by tetramethylpyrazine (TMP). Recently, co-transcription factor YAP1 is reported to control autophagy and is mandatory to stimulate autophagic flux. This study constructed *in vitro* and *in vivo* models using clinically related dosages of AS III. Mitophagy, upregulated YAP1 expression, and Nrf2 activation were observed, with upregulation of p62 representing the occurrence of autophagic flux blockade. In HK-2 cells, oxidative stress induced by AS III promoted sustained Nrf2 activation, which enhanced p62 transcription at an early phase. Subsequently, p62 accumulation induced Nrf2 nuclear translocation, which in turn promoted p62 expression, forming a feedback loop to induce autophagic flux blockade, which was aggravated by the autophagic flux blocker chloroquine (CQ). TMP reversed such processes and protected tubular cells, while silencing YAP1 and Nrf2 attenuated TMP renoprotections. YAP1 agonist PY-60 increased Nrf2 expression, while YAP1 knockdown counteracted it and diminished TMP effect on autophagic flux. Furthermore, blocking Nrf2 caused YAP1 accumulation. CO-IP and immunofluorescence co-localization results confirmed co-nuclear translocations of YAP1 bound to dissociated Nrf2 that induced autophagic flux blockade. In conclusion, the present study identified novel mechanisms that TMP alleviated AI-AKI by improving the autophagic flux blockade via a YAP1-Nrf2-p62-dependent mechanism.

## Introduction

As a naturally occurring toxic metalloid and class I carcinogen, arsenic is a ubiquitous environmental contaminant and a health threat on a global scale 1. According to data from the World Health Organization (WHO), there are approximately 140 million people worldwide who suffer from arsenic pollution in some 50 countries 2. Chronic arsenic exposure could result in cancer of the skin, liver, bladder, kidney, and lung, as well as cardiovascular disease and neurological, developmental, and reproductive abnormalities 3. Despite its adverse health effects, short-term, acute arsenic exposure has clinical therapeutic value in the treatment of acute promyelocytic leukemia (APL) and other solid tumors 4. The kidney is an important excretory organ of arsenic in the human body, so it is also one of the main target organs of arsenic toxicity. Different from the nephrotoxicity caused by long-term, low-dose arsenic exposure in the environment, the potential kidney injury induced by clinical use of AS III as an antineoplastic agent remains largely unclear and controversial. Consistent with this, the main clinical concerns during AS III treatment focus on the side effects on the marrow, liver, and cardiovascular system, not the kidney 5. As reported cases of acute kidney injury (AKI) among APL patients receiving AS III treatment increased gradually 6-8, it is imperative that more attention be given to the potential risk and the mechanisms of kidney damage after AS III exposure in clinical settings.

Autophagy is a vital self-protective mechanism for maintaining cellular homeostasis. Nevertheless, excessive autophagy can result in autophagic cell death, a specific form of programmed cell death 9. In AI-AKI, the activation of autophagy has been shown to alleviate AS III-induced nephrotoxicity, while autophagy inhibition may worsen kidney injury 10. Evaluating autophagic flux provides insights into autophagy levels, as both autophagosome formation and clearance influence its efficiency 11. However, it is unclear whether autophagic flux blockade is involved in AI-AKI lesions and what changes might occur.

YAP1, as a co-transcriptional factor, plays an important role in alleviating AKI, and regulation of YAP1 inhibits renal tubular cell apoptosis, promotes cell regeneration and repair, and attenuates inflammation 12-14. Recently, YAP1 was reported to act as AMPK downstream to control autophagy, and its nuclear exclusion is mandatory to stimulate autophagic flux. Nuclear retention of YAP1 in renal tubular epithelial cells results in impaired autophagic flux 15. However, the mechanism by which YAP1 affects autophagic flux in AI-AKI remains unknown.

TMP, as a bioactive ingredient of the Chinese herb Chuanxiong (Rhizoma Chuanxiong), has several biological functions, such as anti-oxidation, anti-inflammation, and vasodilator effects to name a few 16, 17. Moreover, TMP has been used clinically to treat a variety of kidney diseases as well as cardiovascular and cerebrovascular diseases in China 18. Following up on our previous data in HK-2 cells that AS III exposure activates multiple nuclear transcription factors simultaneously, including Nrf2, NF-κB, and AP-1, interestingly, TMP, or 2, 3, 5, 6-tetramethylpyrazine could protect HK-2 cells against such arsenic nephrotoxicity by reversing the above pathological processes 19, 20. Our previous studies have highlighted the efficacy of TMP in protecting human renal proximal tubular epithelial cells in a preclinical nephrotoxicity model treated with AS III by preventing mitochondrial dysfunction and autophagy.

Therefore, the aim of this study was to investigate the relationship between YAP1 and Nrf2/p62-mediated autophagic flux blockade in TMP amelioration of AI-AKI to explore the underlying mechanisms. The results of the study provide new insights into the improvement of AI-AKI by TMP.

## Materials and methods

All chemicals were purchased from Sigma (St. Louis, Mo., USA) unless otherwise stated.

### Animals and treatment

All research protocols for animal experiments were previously approved by the Animal Welfare and Ethical Use Committee of the Shanghai Municipal Hospital of Traditional Chinese Medicine (2022025). Thirty-two adult male Sprague-Dawley rats (weighing 200-250 g and aged 8-10 weeks) were purchased from the Shanghai Laboratory Animal Research Center (certificate number: 2020-009). All rats were housed in an air-conditioned room at 23 °C with a cycle of 12 h/12 h light/dark, and food and water were provided ad libitum as described previously 21. Initially, 32 rats were randomly divided into 4 groups of 8 animals each: controls (CON), rats injected with AS III (AS III), rats treated with 150 mg/kg/d N-acetylcysteine (NAC) and injected with AS III (AS III + NAC), and rats treated with 80 mg/kg/d TMP and injected with AS III (AS III + TMP). Based on our previously published data, NAC was used as the positive antioxidant control in the current study 19, 20, 22. Sodium arsenite (NaAsO_2_) was given by intraperitoneal injection at 10 mg/kg/d for 10 consecutive days to establish the rat renal injury model. NAC and TMP, both approved clinical medicines in China 23, 24, were obtained from Hainan Zambon Pharmaceutical Co., Ltd. and Zhengzhou Cheuk-Fung Pharmaceutical Co., Ltd. respectively. 150 mg/kg/d NAC and 80 mg/kg/d TMP were injected intraperitoneally 5 days before modeling, once daily for 15 days. Rats in the CON group were administered with the same volume of saline only. In our previous animal and cell experiments, it has been repeatedly shown that these drugs have no obvious toxicity in rats or renal tubular epithelial cells when given at clinically relevant doses 19, 20. Therefore, a separate TMP and NAC control group was not established in the present study. On day 15, 24-hour urine samples were collected in metabolic cages for the determinations of urinary AKI biomarkers, including urinary N-acetyl-glucosaminidase (UNAG) and urinary-glutamyl transpeptidase (UGGT), and all animals were euthanized under pentobarbital (50 mg/kg) anesthesia. Blood samples were collected from the abdominal aorta for the measurement of serum creatinine (Scr) and serum blood urea nitrogen (BUN), and kidneys were removed quickly for biochemical and morphological examinations.

### Cell culture and treatment

The human proximal tubular cell line HK-2 (American Type Culture Collection, Manassas, VA, USA) was grown in culture medium (DMEM/F12 medium + 5 ng/ml epidermal growth factor and 50 μg/ml bovine extract + 100 U/ml penicillin and 100 μg/ml of streptomycin) at 37 °C in a 5% CO_2_ humidified environment. The next stock solutions were prepared: 50 mM sodium arsenite, TMP (50 μM, 100 μM) in PBS, and TMP were added into media 30 min before AS. The concentration and treatment time for PY-60 (MedChemExpress, USA) were based on previous literature 25. Briefly, HK-2 cells were exposed to PY-60 (10 μM) for 24 hours.

### Detections of renal function and urinary AKI biomarkers

Renal function, including serum creatinine (Scr) and serum blood urea nitrogen (BUN), was detected by the sarcosine oxidase method and urease method, respectively. Urinary AKI biomarkers (UNAG and UGGT) were evaluated with an enzyme-linked immunosorbent assay kit according to the manufacturer's instructions (MedicalSystem, Ningbo, China).

### Histological and immunohistochemistry examinations

Tissue samples from the left kidney were fixed in 4% paraformaldehyde and embedded in paraffin for sectioning at 5 μm thickness. Samples were stained with either hematoxylin & eosin (H&E) or immunohistochemistry (IHC) and observed under light microscopy as described previously 21.

### Transmission electron microscopy (TEM)

Right renal cortex samples were sectioned into pieces (1 × 1 mm) on ice and processed using standard histological steps as described in our previously published studies 21. Ultrathin sections were stained and visualized under a H-7500 transmission electron microscope (TEM) (Hitachi, Tokyo, Japan) for ultrastructural analyses and evaluation of mitophagy. Ten intact renal tubular epithelial cells were observed in each section, and the average number of autophagic vacuoles per tubular cell was counted.

### Immunofluorescence staining

Frozen rat kidney cortex tissues were fixed with 4% paraformaldehyde and processed for immunofluorescence staining. Samples were incubated with primary antibodies against p62 (Abcam, UK), YAP1 (Cell Signaling Technology, Danvers, MA), or Nrf2 (Cell Signaling Technology, Danvers, MA), followed by incubation with the Alexa Fluor-conjugated secondary antibodies (Invitrogen). Pre-immune serum was used instead of the primary antibody as a negative control. Fluorescence microscopy (TCS SP8, Leica Microsystems) was used for observation, and samples were processed simultaneously using the same acquisition parameters for assessing fluorescence intensity. Image J software was used for the semi-quantitative analysis of immunofluorescence according to the software instructions. Ten randomly chosen fields per section were analyzed.

### GFP-mRFP-LC3 transfection

The HK-2 cells (2 × 10^5^ cells/well) were seeded onto sterile coverslips in 6-well plates. GFP-mRFP-LC3 vectors were transfected according to the manufacturer's instructions (Hanbio Biotechnology, China), and transfected cells were cultured for 48 h in an environment at 37°C and 5% CO_2_. Subsequently, the cells were treated with 10 μM AS treatment at different time points. The coverslips were washed in cold PBS, mounted onto slides, and inspected under a Leica laser scanning confocal microscope (Leica Microsystems GmbH). Fluorescent puncta were observed in the cells, and the results are presented as the average number of puncta per cell.

### RNA interference and transfection

RNA interference siRNA for Nrf2 (human), YAP1 (human) and SQSTM1 (human) was purchased from RiboBio Co., Ltd. (Guangzhou, China). HK-2 cells were seeded in 6-well plates and transfected with 50 nM siRNA in 2 mL of medium containing jetPRIME transfection reagent (Polyplus, France) according to the manufacturer's protocol. The cells were cultured in a CO_2_ incubator for 24-48 hours. Subsequently, the efficiency of interference in transfected cells was analyzed. The cells were then treated with 10 μM AS and/or 100 μM TMP for 3 hours and 6 hours.

### Molecular docking

The structure files of YAP1 and Nrf2 proteins were obtained from the PDB database, respectively. Subsequently, these proteins were subjected to docking pre-processing using PyMol 2.5.32, and molecular docking was performed using ZDOCK 3.0.21 to predict the binding mode between the proteins. The online tool prodigy3 was used for binding energy assessment of protein complex conformations (https://wenmr.science.uu.nl/prodigy/). Finally, binding modes based on optimal binding energies were visualized and analyzed.

### Co-immunoprecipitation (CO-IP)

The procedure was performed according to the instructions of the Co-Immunoprecipitation Kit (Absin, China). Briefly, protein lysates were collected from HK-2 cells using 500 μl lysis buffer, incubated on ice, and then fragmented by sonication. The supernatant after centrifugation was incubated with anti-YAP1 antibody, anti-Nrf2 antibody, or control IgG at 4 °C overnight, followed by incubation with 5 μl protein A/G agarose beads at 4 °C overnight. After centrifugation, the precipitate was collected, washed three times with 1× washing buffer, resuspended in 1× SDS sample buffer, and analyzed by western blotting.

### Immunofluorescence co-localization

Cells were processed, fixed in 4% paraformaldehyde, treated with 0.1% Triton-X, blocked with 5% BSA, and subsequently incubated with antibodies against Nrf2 and YAP1 (Proteintech, China, 1:300) from different species sources. Following incubation with CoraLite488 or CoraLite647 (Proteintech, China, 1:200) fluorescent secondary antibodies, nuclei were stained with DAPI (Beyotime Biotechnology, China) and imaged using a laser confocal microscope (TCS SP8, Leica Microsystems). The spatial positions of the fluorescence were observed for overlap to determine co-localization.

### Western blotting analysis

Western blotting analysis for rat kidney cortex tissue was conducted as described previously 19. Cytoplasmic and nuclear proteins from HK-2 cells were extracted following the manufacturer's protocol (Beyotime Biotechnology, China). First, cytoplasmic proteins were isolated using a cytoplasmic extraction reagent. The remaining supernatant was carefully removed to avoid contamination of the nuclear fraction. Subsequently, a nuclear extraction reagent was added, and the mixture was vigorously shaken for 30 minutes. After centrifugation, the supernatant was collected as the nuclear protein fraction. Image J software was used for density analyses of the results. The specific primary antibodies included the following rabbit antibodies: anti-YAP1 (Abcam, UK), anti-Nrf2 (Abcam, UK), anti-p62/SQSTM1 (Abcam, UK), anti-LC3B (Cell Signaling Technology, Danvers, MA), and mouse anti-GAPDH (Sigma).

### Statistical analysis

All experiments were repeated independently for a minimum of three times and results were expressed as means ± SD. All comparisons were performed by either one-way ANOVA or a two-tailed t-test analysis depending on the number of conditions compared in each experiment. One-way ANOVA was followed by Tukey's post hoc test. *P*<0.05 value was considered significant.

## Results

### TMP attenuates AI-AKI by improving the autophagic flux blockade in rats

**Figure 1:**
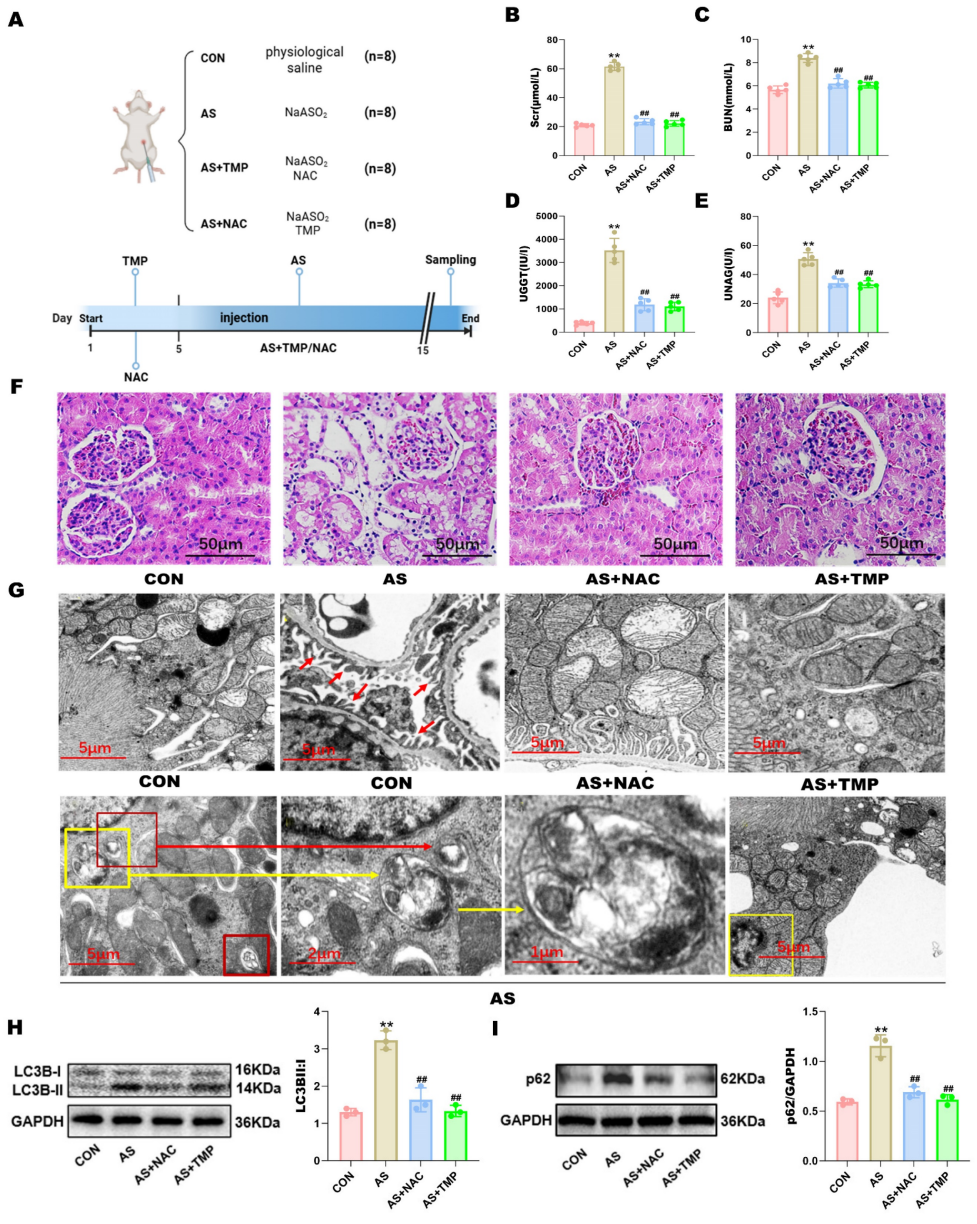
**TMP inhibits AI-AKI in rats. (A)** Establishment of animal models. **(B)** The levels of serum creatinine. **(C)** The levels of serum blood urea nitrogen. **(D)** The levels of urinary γ-glutamyl transpeptidase. **(E)** Urinary N-acetyl-β-glucosaminidase were examined by automated biochemistry assays. Data are shown as means ± SD. Figures are representative of 5 rats from each group. **(F)** Pathological changes of the kidney tissues by HE staining (scale bar = 50µm). **(G)** Autophagy and mitophagy in tubular epithelial cells were increased in AS rats and suppressed by TMP pretreatment. Transmission electron microscopy (TEM) images of the kidney in different groups. Red arrows in the CON group indicate the normal glomerular basement membrane and podocytes in rats (scale bar = 5 µm). Autophagosomes (red frames in AS 1, indicative of autophagy) and mitochondria encapsulated in autophagosomes in AS III rat (yellow frame in AS 1 and AS 2, indicative of mitophagy) (×9000). (AS 3) Higher magnification TEM of (AS 2) (×16000). The yellow frame in (AS 4) indicates tubular cell undergoing apoptosis in AS rats as characterized by the condensation of nuclear chromatin. **(H)** Autophagy-associated protein LC3B was detected by western blotting method. **(I)** Western blotting analysis of p62 (**P<0.01 versus CON, ^##^*P*<0.01 versus AS, *n*=3).

Fluent autophagic flux in renal tubular cells plays a protective role in AKI, while blockade of autophagic flux exacerbates kidney injury. To investigate whether blockade of autophagic flux occurs in AI-AKI, this study constructed *in vivo* and* in vitro* models using clinically related dosages of AS III. TMP pretreatment significantly suppressed the levels of Scr, BUN, UNAG, and UGGT compared with the AS group, indicative of their renal protective effects (Fig. 1A-E). Corroboratively, pathological analysis by H&E staining demonstrated severe renal tubular injury in AS III-treated rats, as evidenced by vacuolar degeneration in both proximal and distal tubular cells, increased epithelial cell shedding, and disruption of tubular brush border (Fig. 1F). Similarly, such tissue damage was suppressed by pretreatment with TMP.

In order to ascertain the presence of autophagy in renal tubular cells after AS III exposure for 14 days, ultrastructural analyses by TEM were performed, which is often considered the gold standard. As shown in Fig. 1G, elevated renal tubular autophagy in kidneys from AS III-treated rats was characterized by the higher quantities of autophagosomes (indicated by yellow or red frames) and double or multiple membrane-encapsulated structures compared to the CON group. Additionally, ultrastructural analysis revealed nuclear chromatin condensation and nuclear membrane wrinkling in renal tubular cells consistent with apoptotic features (the right yellow frame in Fig. 1G). Concomitantly, AS III exposure induced the encapsulation of mitochondria in autophagosomes (indicated by the yellow frame and yellow arrow in the AS group). These results provided strong evidence that mitophagy is involved in the pathogenesis of AI-AKI. Furthermore, TMP pretreatment markedly decreased the quantity of autophagosomes and the degree of mitophagy observed and reduced the abundances of LC3B-II (Fig. 1H). In addition, elevated p62 expression indicated the occurrence of autophagic flux blockade (Fig. 1I).

### TMP improves AS III-induced autophagic flux blockade *in vitro*

**Figure 2:**
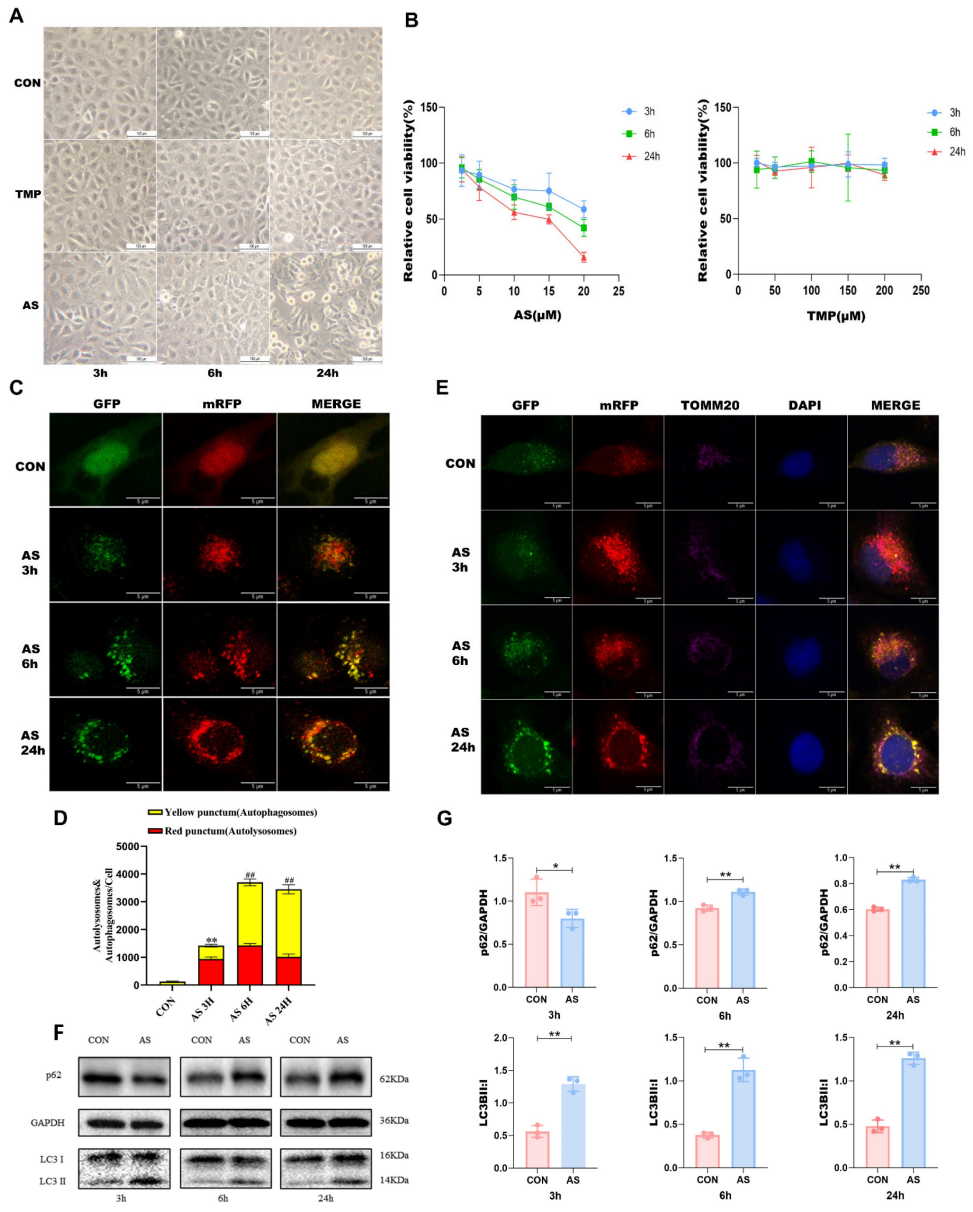
** AS III results in autophagic flux blockade in HK-2 cells. (A)** Changes in cell morphology at different time points were observed under an original 100-fold magnification. **(B)** Cell viability was measured by CCK8 assay. **(C)** Observation of mRFP-GFP-LC3 fluorescence signal position (scale bar = 5µm). **(D)** Quantitative analysis of the autophagosomes (yellow dots) and autolysosomes (red dots). The fluorescence intensity of autophagosomes (yellow dots) and autolysosomes (red dots) in each cell was compared by using ImageJ software. **(E)** After staining with mitochondrial membrane proteins, co-localization of mitochondria and LC3 was performed (scale bar = 5 µm). **(F&G)** Western blotting analysis of p62 and LC3B at different time points (*P<0.05, **P<0.01 versus CON, *n*=3).

As the duration of cellular arsenic exposure increased, progressive phenomena such as cellular shrinkage, membrane rupture, and cell death were observed (Fig. 2A). To ascertain the status of autophagic flux, we employed mRFP-GFP-LC3 labeling to visualize intracellular LC3 and monitor its changes. Following a 3-hour AS treatment, a substantial increase in red puncta within the cells was observed, indicating a robust autophagic flux (Fig. 2C and D). Conversely, the proportion of red puncta gradually diminished after 6 hours and 24 hours of treatment, confirming the blockade of autophagic flux—a finding consistent with our Western blotting results. In addition, we labeled the mitochondrial membrane protein TOM20 and conducted co-localization analysis of mitochondria and LC3 through laser confocal microscopy. The purple color in the figure represented mitochondria (Fig. 2E). Notably, the location of autophagic activity overlapped with that of mitochondria, implying that the observed autophagy was mitophagy. This finding aligns with the outcomes of our animal experiments. Following a 3-hour exposure of HK-2 cells to AS III, we observed a reduction in p62 expression and an elevation in the LC3B II/I ratio, indicating the occurrence of autophagy. Conversely, after 6 and 24 hours of AS III treatment, there was an augmentation in the p62 levels. Furthermore, the transition from LC3B I to II remained higher than that observed in the CON group (*P*<0.01), suggesting a progressive accumulation of p62. These results suggested that AS III exposure induced autophagic flux blockade *in vitro* (Fig. 2F and G).

**Figure 3:**
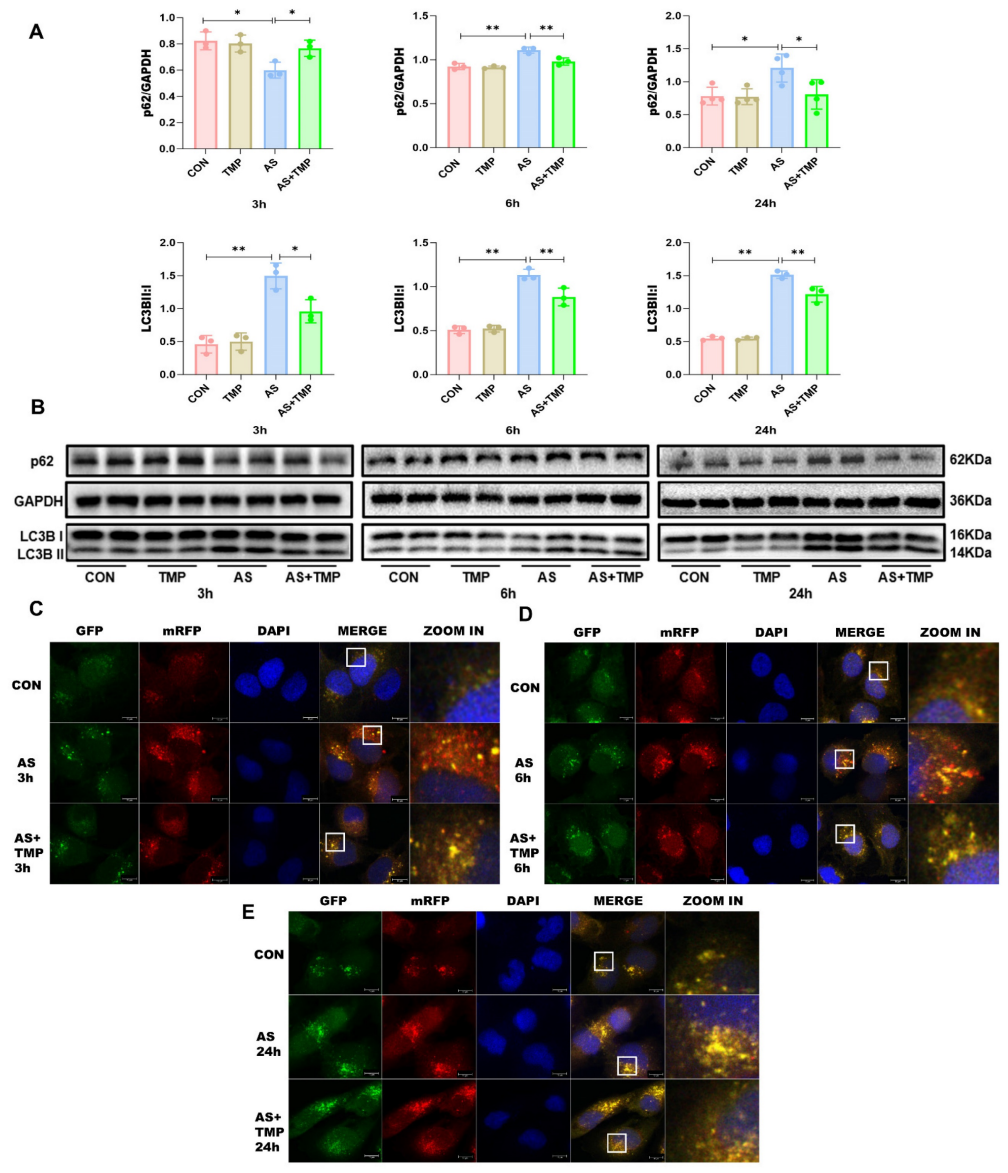
** TMP inhibits AS III-induced autophagic flux blockade in HK-2 cells. (A)** Relative densitometry analysis of the ratio of p62 and LC3 to GAPDH. **(B)** Cells were treated with AS (10μM) or TMP (100μM) at different time points, followed by Western blot analysis (*P< 0.05, **P<0.01, *n*=3). **(C-E)** Observation of mRFP-GFP-LC3 fluorescence signal position at different time points (scale bar = 10µm).

**Figure 4:**
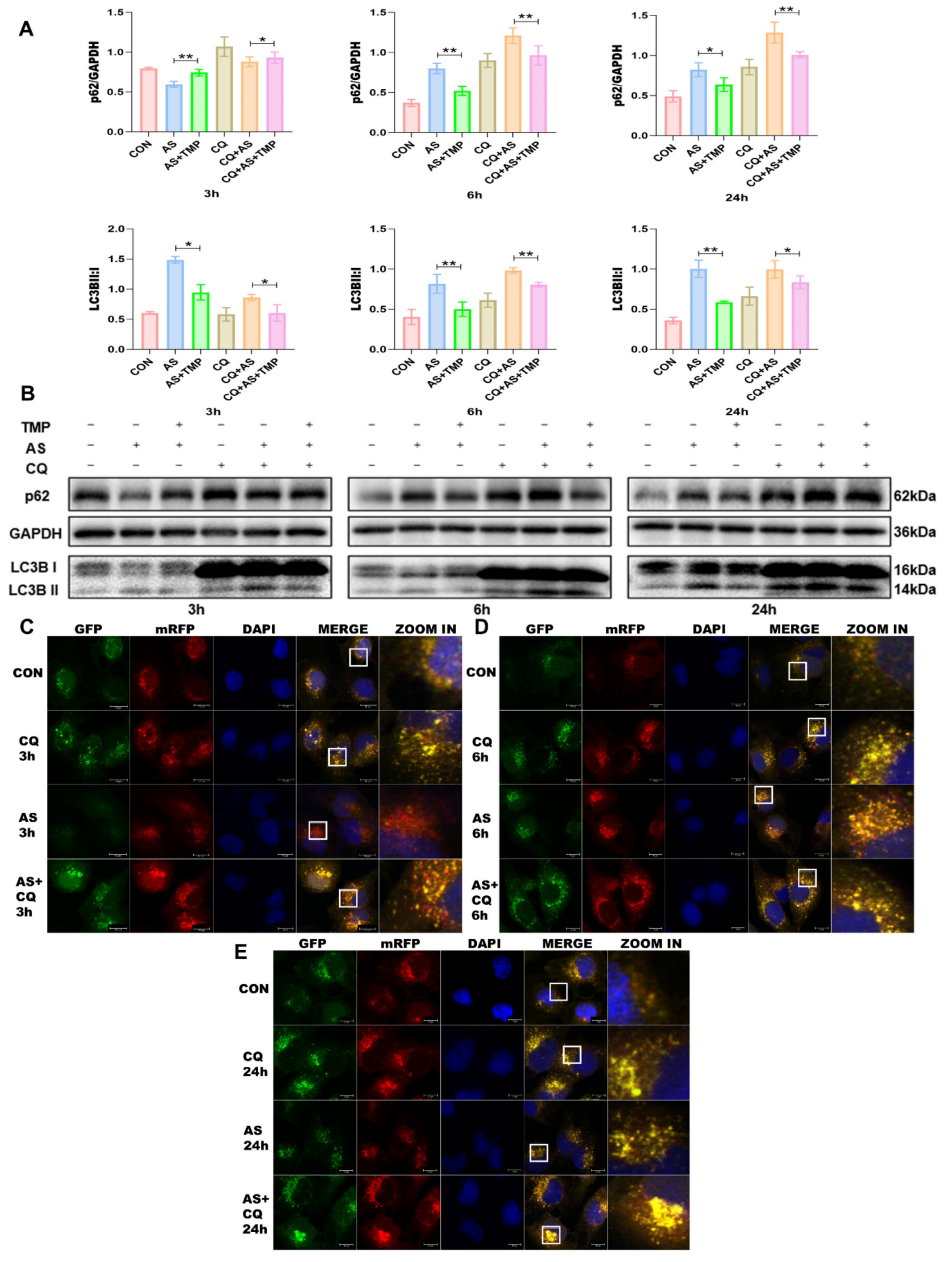
** TMP partially restores CQ-induced autophagic flux blockade. (A)** Relative densitometry analysis of the ratio of p62 and LC3 to GAPDH. **(B)** Cells were treated with AS (10μM) or TMP (100μM) or CQ (50μM) at different time points, followed by Western blot analysis (*P<0.05, **P<0.01, *n*=3). **(C-E)** Observation of mRFP-GFP-LC3 fluorescence signal position at different time points (scale bar =10µm).

Following 3h of AS exposure, TMP pretreatment resulted in a reduced level of cellular autophagy, with autophagic activity approaching that of the CON group, which helped cells withstand the oxidative stress induced by arsenic toxicity. Subsequently, the autophagic flux of the cells was blocked at 6h and 24h (Fig. 3A-E), and the accumulation of p62 would lead to autophagic dysfunction; damaged organelles within cells caused by oxidative stress couldn't be efficiently removed. TMP-pretreated cells could partially rescue the autophagic blockade to mitigate the damage, thus manifesting a protective role on cell morphology and function. Furthermore, we added the autophagy inhibitor chloroquine (CQ). After 3 hours of AS III exposure, CQ counteracted AS III-induced autophagy while co-blocking the autophagic flux at 6h and 24h. Blockade of autophagic flux by CQ significantly enhanced AS III-induced renal injury, whereas TMP could restore autophagic flux for therapeutic effect (Fig. 4A-E).

### TMP inhibits AS III-induced autophagic flux blockade by inhibiting the YAP1-Nrf2-p62 pathway

**Figure 5:**
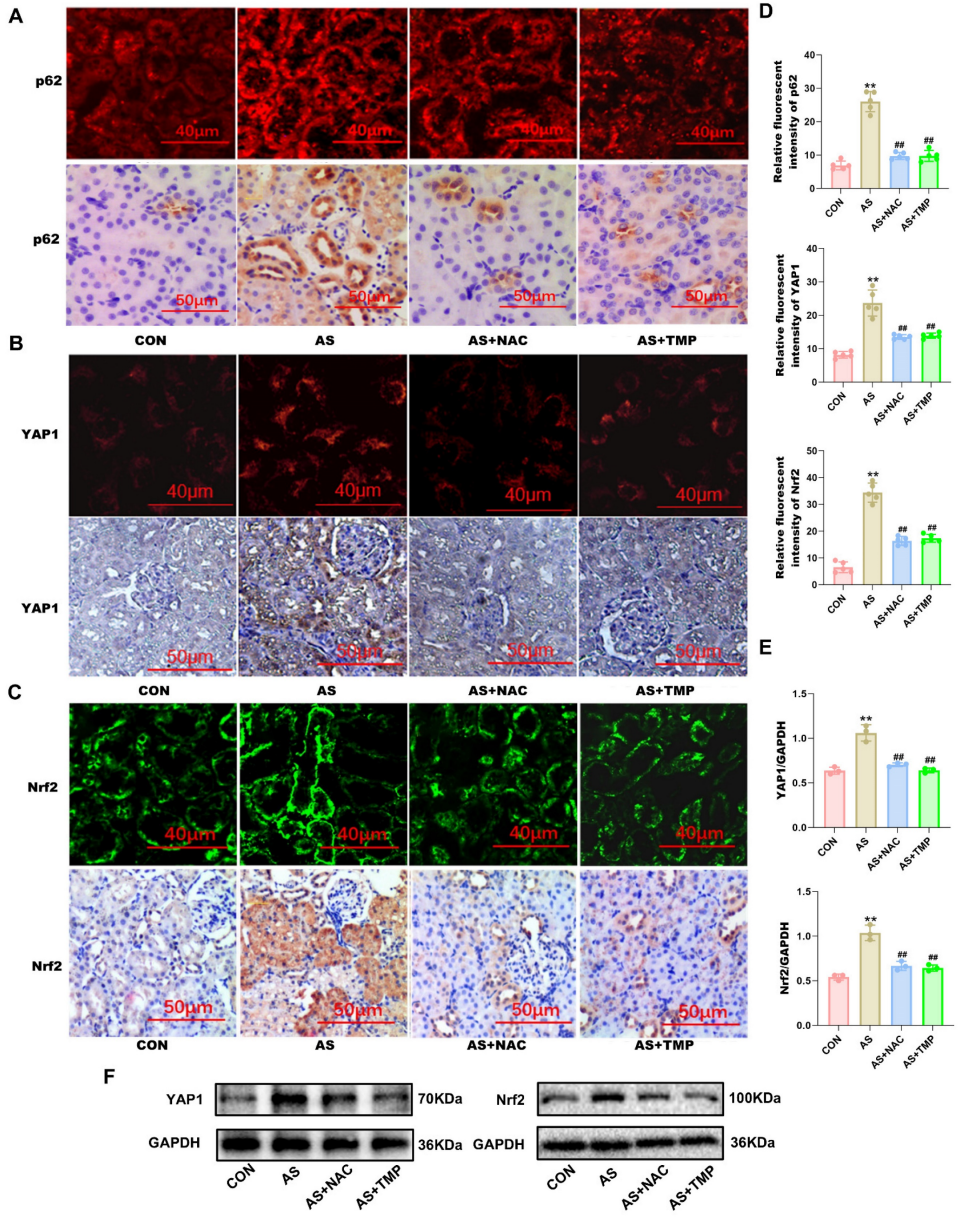
** TMP inhibits YAP1-Nrf2-p62 pathway in rats. (A)** AS III-induced p62 activation was verified by increased level of p62 expression in renal tubule, which were inhibited by TMP pretreatment. p62 expression was assessed by immunofluorescence (scale bar =40µm) and IHC staining (scale bar =50µm) in rat kidney tissues. **(B)** YAP1 expression was assessed by immunofluorescence (scale bar =40µm) and IHC staining (scale bar =50µm). **(C)** Nrf2 expression was assessed by immunofluorescence (scale bar =40µm) and IHC staining (scale bar =50µm). **(D)** Semiquantitative analysis of immunofluorescence staining for p62, YAP1, and Nrf2. Figures are representative of 5 rats from each group. Data are represented as mean ± SD (**P<0.01 versus CON. ^##^*P*<0.01 versus AS,* n*=5). **(E&F)** Western blotting analysis of YAP1, and Nrf2. (**P<0.01 versus CON. ^##^*P*<0.01 versus AS,* n*=3).

To clarify the renoprotective mechanism of TMP in alleviating AI-AKI, we investigated its effects on the YAP1-Nrf2-p62 pathway and the associated autophagic flux blockade. Immunofluorescence, immunohistochemistry, and western blot results all indicated that ASIII exposure activated the YAP1-Nrf2-p62 pathway *in vivo* (Fig. 5A-F).

**Figure 6:**
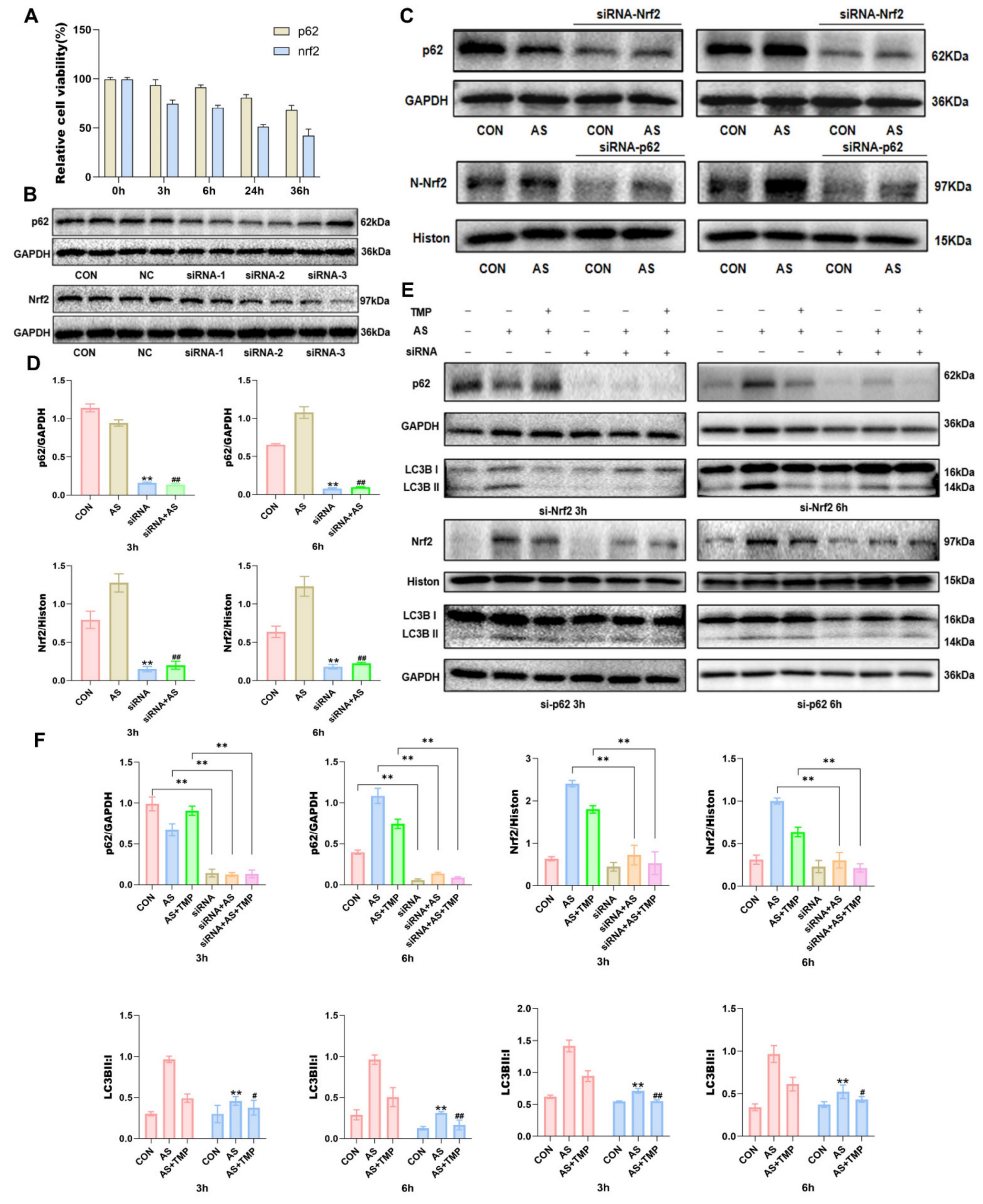
** TMP attenuates AI-AKI by modulating the Nrf2/p62/Nrf2 feedback loop. (A)** Cell viability was measured by CCK8 assay after transfection with Nrf2 and p62 siRNA. **(B)** Knockdown efficiency was evaluated after successful siRNA transfection for 24 hours. Three different sequences were available for each siRNA, and the two most effective ones were selected for subsequent experiments. NC: Negative Control, PC: Positive Control. **(C&D)** Western blot for Nrf2 and p62 in HK-2 cells exposed to AS (10μM) or siRNA (50nM) at different time points. **P<0.01 versus CON, ^##^*P*<0.01 versus AS, *n*=3. **(E)** After knocking down Nrf2 and p62, the expression of p62 and levels of Nrf2 within the cell nucleus were measured. **(F)** Quantification of protein levels determined with Western blot. Upper: *P< 0.05, **P<0.01 compared with CON (or AS and AS+TMP); Lower: **P<0.01 compared with AS III, ^#^*P*<0.05, ^##^*P*<0.01 versus AS+TMP, *n*=3. Representative image: Western blot for Nrf2, P62 and LC3B in HK-2 cells exposed to AS (10μM) or TMP (100μM) or siRNA (50nM) at different time points.

However, pretreatment with TMP inhibited the activation of the YAP1-Nrf2-p62 pathway, alleviated the autophagic flux blockade caused by p62 accumulation, and attenuated AI-AKI. Knocking down Nrf2 or p62 reduced the cellular antioxidant activity, as evidenced by the results of the CCK-8 assay (Fig. 6A). The results indicated a close association between the Nrf2-p62 signaling pathway and cell survival. Previous studies have shown that oxidative stress triggers sustained activation of Nrf2, impairing p62 degradation via the autophagic pathway 26. The resulting accumulation of p62 further promotes Nrf2 nuclear translocation, forming a feedback loop and ultimately leading to a blockade of autophagic flux. The results demonstrated a noteworthy reduction in the Nrf2 expression in the nucleus where p62 was knocked down, as well as a distinct decrease in p62 expression in cells following Nrf2 knockdown (Fig. 6C and D). This suggested that knocking down Nrf2 and p62 expression inhibited the role of this feedback loop in autophagic flux regulation. Furthermore, knockdown of both Nrf2 and p62 led to an overall reduction in cellular autophagy level. The protective effect of TMP on autophagic flux was also significantly weakened after knockdown compared with the TMP-pretreated group (Fig. 6E and F).

**Figure 7:**
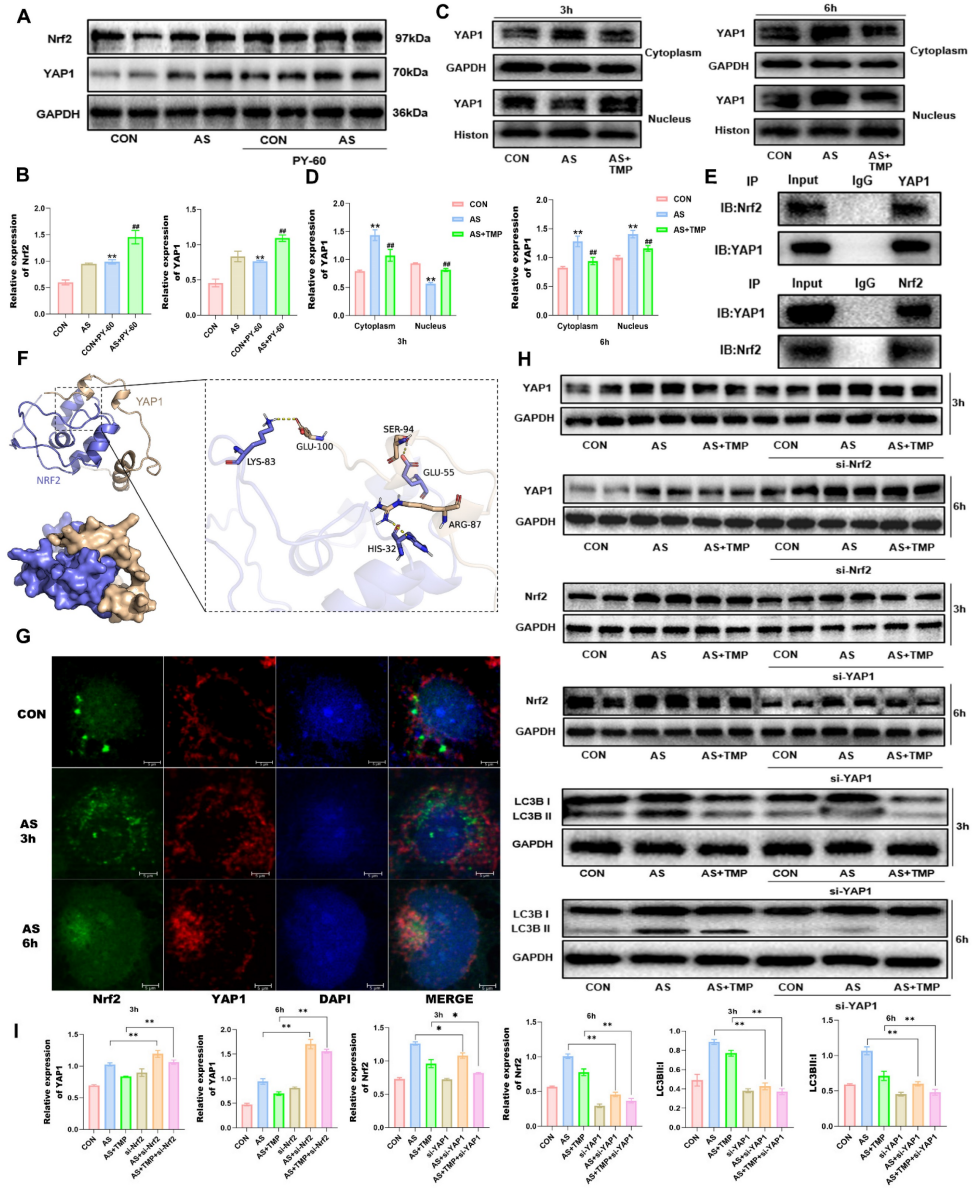
** TMP alleviates AI-AKI by improving the autophagic flux blockade via YAP1-Nrf2 pathway. (A)** Effects on AS III-exposed HK-2 cells after YAP1 agonist. **(B)** Quantification of protein levels determined with Western blotting. (**P<0.01 versus CON,^ ##^*P*<0.01 versus AS, *n*=3). **(C&D)** Western blotting analysis of YAP1 in cytoplasm and nucleus at different time points under ASIII exposure. (**P<0.01 versus CON,^ ##^*P*<0.01 versus AS, *n*=3). **(E)** Co-immunoprecipitation results of YAP1 and Nrf2. **(F)** Molecular docking results of YAP1 and Nrf2. Hydrogen-bonding interactions between GLU-100, SER-94, and ARG-87 on the YAP1 protein and LYS-83, GLU-55, and HIS-32 on the Nrf2 protein are their possible binding sites. **(G)** Immunofluorescence co-localization of YAP1 and Nrf2 at different time points (scale bar = 5µm). **(H&I)** Western blotting analysis of Nrf2 knockdown on YAP1 expression, and effect of YAP1 knockdown on Nrf2 and LC3B expression after exposure to ASIII at different time points. (*P<0.05, **P<0.01 compared with AS or AS+TMP, *n*=3).

Moreover, nuclear retention of YAP1 in renal tubular epithelial cells under stress leads to autophagic flux blockade 15. PY-60, an agonist of YAP1, increased the expression of Nrf2 under arsenic exposure conditions, and YAP1 may be an upstream signaling molecule for Nrf2 (Fig. 7A and B). Early during AS III exposure, total YAP1 expression increased, with higher levels observed in the cytoplasm compared to the nucleus (Fig. 7C and D). After 6 hours of arsenic exposure, nuclear YAP1 expression significantly rose, indicating enhanced nuclear translocation, which coincided with the nuclear translocation of Nrf2. To further investigate the potential interaction between YAP1 and Nrf2, immunoprecipitation and molecular docking analyses were performed. Results indicated multiple binding sites for YAP1 and Nrf2, which could be located at LYS-83, GLU-55, HIS-32, and other positions of Nrf2 (Fig. 7E and F). AS III exposure led to a progressive accumulation of p62, which promoted the simultaneous nuclear translocation of YAP1 and Nrf2. Their spatial localization overlapped, coinciding with the timing of autophagic flux blockade, as confirmed by immunofluorescence co-localization results (Fig. 7G). The results showed that knockdown of YAP1 reduced the expression of Nrf2, while knockdown of Nrf2 led to the accumulation of YAP1 (Fig. 7H). Knockdown of YAP1 reduced autophagy levels, and the role of TMP in alleviating autophagic flux blockade was also significantly attenuated (Fig. 7H and I), suggesting that the role of TMP in regulating autophagic flux is dependent on the YAP1-Nrf2-p62 pathway. Taken together, TMP attenuates AI-AKI by improving the autophagic flux blockade via a YAP1-Nrf2-p62-dependent mechanism.

## Discussion

Initially, studies of AS III toxicity focused on QTc prolongation, hepatotoxicity, and leukemia 5. In recent years, with increasing reports of AKI in patients following AS III treatment, the issue of clinical dose AS III treatment-related kidney injury is becoming a new concern, supported by clinical case reports 1, 6-8. In this study, we identified autophagic flux blockade in mitochondria in renal tubular cells. TMP exerts a nephroprotective effect by repairing the impaired autophagic flux via a YAP1-Nrf2-p62-dependent mechanism.

Autophagy, as a crucial mechanism for maintaining cellular homeostasis and controlling protein and organelle quality, remains controversial regarding whether it plays a protective or damaging role in AKI 27. Autophagy is also a crucial protective mechanism for organ clearance of heavy metal toxicity 2. Under cellular stress conditions, autophagy has been demonstrated to be a stress response of renal tubular cells to acute injury, potentially serving as a mechanism for enhancing cell survival and self-protection. Autophagic flux is a reflection of the strength of autophagy, and a smooth autophagic flux contributes to the repair of cellular damage, but the role it plays in AI-AKI has not been fully elucidated. In this study, we treated cells with the autophagy inhibitor CQ. Based on cell viability and morphological observations, autophagic flux blockade exacerbated AS III-induced cell damage (Fig. 5B). Studies have shown that in cisplatin-induced AKI, pharmacological inhibition of autophagic flux aggravated injury, whereas autophagy activation had a protective effect 28. Nonetheless, dysregulated autophagy can also result in cell death. Additionally, increasing evidence suggests that YAP1 activates autophagic components transcriptionally or promotes the active assembly of autophagic complexes through interactions, and that inhibition of the YAP1/autophagy axis impairs autophagic flux, making cells more susceptible to apoptosis 29-31.

TMP exerts anti-inflammatory and antioxidant effects by scavenging oxygen free radicals, thereby alleviating oxidative stress. Moreover, it has demonstrated beneficial nephroprotective effects in various acute kidney injuries (AKIs), including those induced by medications, sepsis, and ischemia-reperfusion 21, 32, 33. TMP is widely used as a clinical drug in China for the treatment of various conditions, including myocardial infarction, cerebral infarction, and diabetic nephropathy 18, 34. Our previous findings indicated that the nephroprotective efficacy of TMP in a preclinical model of AS III-induced nephrotoxicity was mediated by preventing mitochondrial dysfunction, inhibiting mitochondrial ROS production, modulating autophagy, and suppressing pro-inflammatory signaling pathways in renal tubular epithelial cells 19, 20. In our current study, TMP was shown to modulate the YAP1-Nrf2-p62-mediated autophagic flux blockade to inhibit AI-AKI.

Based on the present data, we confirmed that autophagic flux blockade in renal tubular epithelial cells plays a critical role in the pathogenesis of AI-AKI. In this study, alterations in mitophagy and autophagic flux within mitochondria were observed, providing further insight into the underlying mechanisms (Fig. 1-5). There is evidence that mitophagy may play a crucial role in AKI 35. Since the gold standard in determining mitophagy is to identify autophagosomes with cup-shaped membranes and other related subcellular structures by electron microscopy 36, we confirmed the presence of such structures in renal proximal tubular cells. The interaction between Nrf2 and p62 plays a crucial role in the blockade of autophagic flux 37. Arsenic is an activator of Nrf2, which is considered one of the protective factors against arsenic-induced toxicity via scavenging free radicals. Under homeostasis conditions, Keap1, as the endogenous inhibitor of Nrf2, binds to Nrf2 to form the Keap1/Nrf2 complex and retains Nrf2 in the cytoplasm 38. In contrast, under physiological stress, the Keap1/Nrf2 complex dissociates and promotes Nrf2 activation and translocation to the nucleus for transcriptional activation of downstream target genes, such as HO-1 39. In addition to Keap1, published data suggested that the level and degradation of active Nrf2 might also be regulated by autophagy and p62 signaling 40. Recently, the positive feedback loop between p621 and Nrf2 was confirmed in a cisplatin-induced AKI model 41. Consistent with these findings, our present study also found that AS III exposure not only induced high expression of Nrf2, but also led to the upregulation of p62. Most likely, the large increase in p62 level observed in the present rat renal injury model could result in the creation of a positive feedback loop to induce persistent activation of Nrf2. Interestingly, p62, as a multifunctional cytoplasmic protein, could act as the receptor of mitophagy 42. We thus speculated that the Nrf2/p62 feedback loop is also the crucial switch in regulating AS III-induced mitophagy and autophagic flux.

YAP1, a key effector molecule of the Hippo pathway, plays an important role in mediating epithelial cell regeneration during renal recovery in AKI 14. Sustained activation of YAP1 upregulates the expression of monocyte chemotactic protein 1 (MCP-1), exacerbating macrophage infiltration and inflammatory responses in AKI 43. In addition, activation of YAP1 could enhance the degradation of autophagic lysosomes to increase autophagic flux 44. Both YAP1 and Nrf2 are activated to enhance cellular self-defense under stress 45, 46. It has been reported that nuclear retention of YAP1 in renal tubular epithelial cells under stress leads to autophagic flux blockade 15, and the results of the present study were consistent with it. In this study, accumulation of p62 led to nuclear translocation of Nrf2, which also resulted in autophagic flux blockade. Immunoprecipitation and immunofluorescence co-localization results indicated that nuclear translocation of YAP1 upon binding to Nrf2 resulted in the blockade of autophagic flux. Interestingly, knockdown of YAP1 reduced Nrf2 expression, whereas Nrf2 was upregulated after TMP pretreatment. This suggested that in the absence of YAP1, TMP pretreatment could not effectively alleviate the oxidative stress state of the cells, and the nephroprotective effect was significantly attenuated. In addition, the role of TMP in alleviating autophagic flux blockade to increase autophagic flux was also notably weakened after Nrf2 knockdown, and the nephroprotective effect of TMP was dependent on YAP1 and Nrf2. In conclusion, TMP may attenuate AI-AKI by improving the autophagic flux blockade through a YAP1-Nrf2-p62-dependent mechanism.

Therefore, TMP might provide a novel choice for the treatment of renal injury induced by a clinical dose of AS III, but the renoprotective mechanism of TMP has not been fully clarified. Finally, we need to further elucidate and define other potential autophagy pathways under this pathological condition.

## Conclusion

In conclusion, we confirmed that autophagic flux blockade in renal tubular epithelial cells is a key factor in the pathogenesis of AI-AKI. TMP mitigated AI-AKI by restoring autophagic flux blockade. The nuclear translocation upon binding of YAP1 and Nrf2, along with the accumulation of p62, jointly contributed to the autophagic flux blockade. TMP may alleviate AI-AKI by improving the autophagic flux blockade via a YAP1-Nrf2-p62-dependent mechanism. Thus, our study improves the understanding of the protective effect of TMP against AS III nephrotoxicity and may help to expand the clinical application of TMP in AKI.

## Figures and Tables

**Figure 8:**
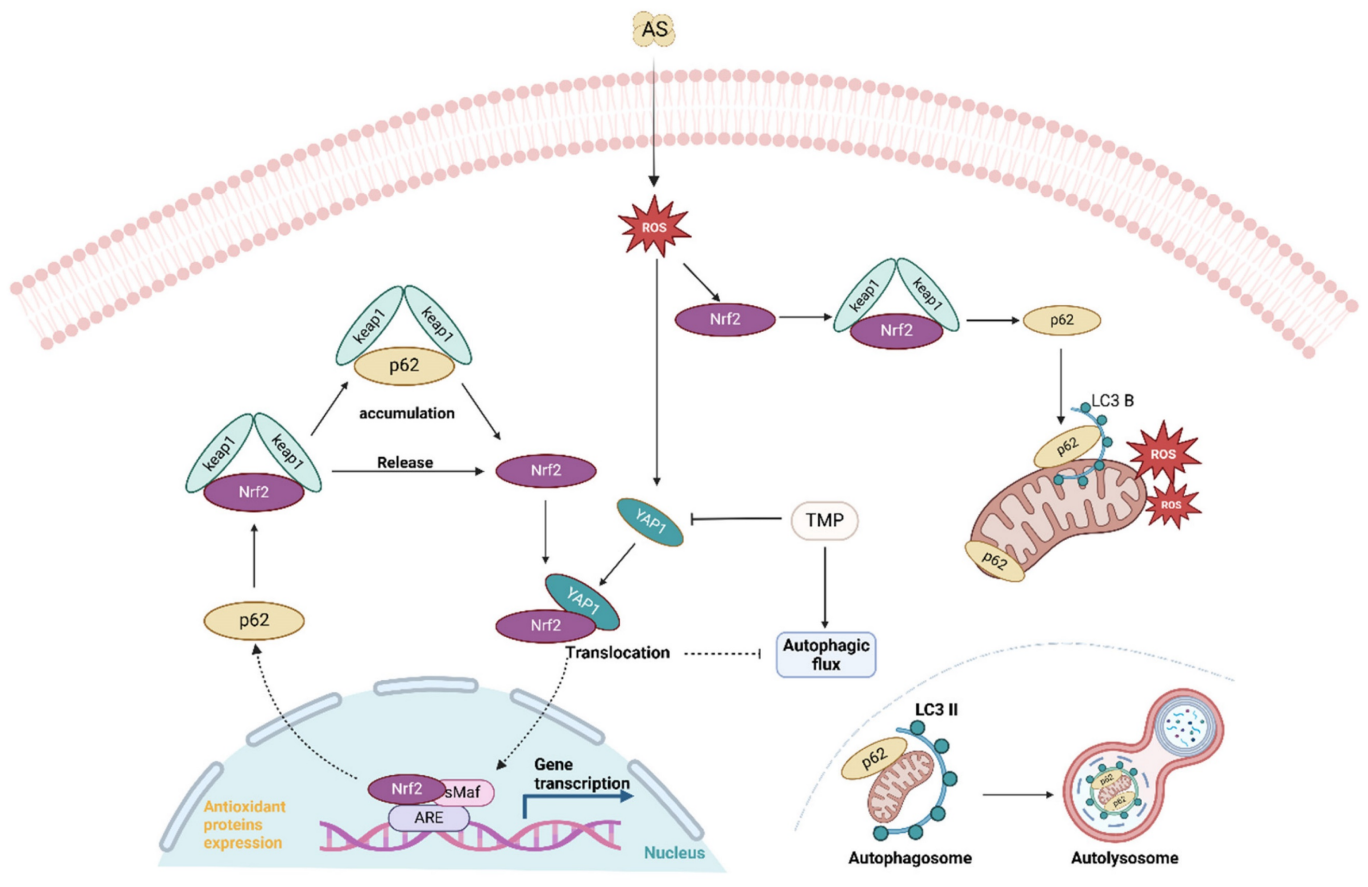
AS III induces autophagy and activates the nuclear translocation of Nrf2, resulting in the accumulation of p62 and autophagic flux blockade. AS III disrupts the Nrf2/p62/Nrf2 feedback loop and activates the YAP1-Nrf2-p62 signaling pathway, contributing to autophagic flux blockade. TMP mitigates autophagic flux blockade through a YAP1-Nrf2-p62-dependent mechanism, thereby alleviating AI-AKI.

## Abbreviations

YAP1: Yes1-associated transcriptional regulator
AS III: sodium arsenite
AI-AKI: ASIII-induced acute kidney injury
ROS: reactive oxygen species
TEM: transmission electron microscopy
CQ: chloroquine
Nrf2: Nuclear factor erythroid 2-related factor 2
Keap1: KEAP1 kelch like ECH associated protein 1
Mfn2: mitofusin 2
AP-1: Activator protein 1
APL: acute promyelocytic leukemia
TMP: tetramethylpyrazine
NF-κB: Nuclear factor kappa-B
NAC: N-acetylcysteine
UNAG: urinary N-acetyl-glucosaminidase
UGGT: urinary-glutamyl transpeptidase
Scr: serum creatinine
BUN: blood urea nitrogen
HO-1: Heme Oxygenase-1
MCP-1: monocyte chemotactic protein 1
